# Risk for Depression, Burnout and Low Quality of Life Among Personnel of a University Hospital in Italy is a Consequence of the Impact One Economic Crisis in the Welfare System?

**DOI:** 10.2174/1745017901713010156

**Published:** 2017-10-13

**Authors:** MG Carta, A Preti, I Portoghese, E Pisanu, D Moro, M Pintus, E Pintus, A Perra, S D’Oca, M Atzeni, M Campagna, E Fabrici Pascolo, F Sancassiani, G Finco, E D’Aloja, L Grassi

**Affiliations:** 1 Department of Health Sciences and Public Health, University of Cagliari, Cagliari, Italy; 2 School of Psychiatric Reabilitation Tecnicians, University of Trieste, Trieste, Italy; 3 Department of Biomedical and Specialty Surgical Sciences, University of Ferrara, Ferrara, Italy

**Keywords:** Depression, Quality of life, Burnout, Economic crisis, Self-report, Public health

## Abstract

**Background:**

Research literature suggests that burnout, depression, and a low mental quality of life (QOL) are common among health care workers. Economic crisis might have increased the burden of burnout, depression and low QOL in health care workers.

**Objectives:**

To identify depression risk, burnout levels, and quality of life in a sample of workers of an Italian university hospital.

**Method:**

Cross sectional study with comparison with two community surveys database results (n = 2000 and 1500, respectively). Overall, 522 workers accepted to take part in the study, representing a 78% response rate (out of 669 individuals).

**Results:**

The frequency of positivity at the screener for Major Depressive Disorder among health care workers was more than double than that in the standardized community sample (33.3% vs 14.1%, p<0.0001). All professionals, except the administrative staff and technicians (*i.e.* those who do not have contact with patients), showed a statistically higher frequency of positivity for depressive episodes compared to the controls. Among the medical staff, the highest risk was found in the surgeon units, while the lowest one was in the laboratories. Surgeons also were those most exposed to high risk of burnout, as measured by the Maslach Burnout Inventory.

**Conclusion:**

Since burnout is linked to patient safety and quality of patient care, and contribute to medical errors, dedicated interventions aimed at reducing poor mental health and low quality of life in medical staff are indicated.

## 1 INTRODUCTION

The current financial crisis has heavily impacted the public sector in Europe and, to a greater extent, in countries in which the crisis was more severe and in those where the public system has higher costs. According to the WHO, “the global financial crisis that began in 2007 can be classified as a health system shock” [1].

In this context, Italy is an interesting case study. Italy has a widespread national public health system, in which, the need for reducing the high level of public deficit and debt led to the so-called “block of personnel turnover” policies, limiting Italian public sector in hiring new workers. At the same time, another consequence of the financial crisis is the continuous raising by law of the eligibility age for pension. Therefore, everyone who has a chance (in terms of age) to retire, they do it to avoid a change that could hamper their opportunity window. In fact, workers are threatened with a possible future political reform of the age for retirement, thus they retire even if they feel able to continue to work.

The result of this financial crisis is that Italy is facing a significant aging process in its workforce. Workers are forced to stay longer at work than they expected or desired. At the same time, workers from the public health sector are highly exposed to a number of job stressors, ranging from work overload, time pressures, low support and lack of role clarity. Research literature suggests that burnout, depression, and a low mental quality of life (QOL) are common among health care workers [2-7]. Further evidence showed that employee’s mental health may affect significantly quality of care, job satisfaction, intent-to-leave and increase the risk of suicidal ideation [8-14]. The public health care workers of the university system live the crisis to a greater extent. The health care workers who teach or operate in the medical faculties of public universities in Italy, unlike other European countries, receive only one salary for two jobs. They really should use a proportion of their working time for a job (treating patients) and a proportion for the other (teaching and research or support teaching and research). The present crisis in the two sectors can double, in this case, its impact on workers.

The main purpose of this study was to identify employees’ depression risk, burnout levels and quality of life, in a sample of workers of an Italian university agency (that manage two University Hospitals). The results were compared with data from community samples. Within the sample of workers, we analyzed the risk of burnout and the socio-demographic determinants and the associated factors related to working conditions (work history, type of department, type of work).

## 2 METHODS

### 2.1 Design

Cross sectional study with comparison with two community surveys database results.

### 2.2 Sample

A sample of 1/3 of the overall staff working in an Italian Public Health University Agency was randomly selected from the staff lists. The research involved a total of 12 clinical units of two hospitals of the Health Agency plus a thirteenth group formed by administrative staff.

### 2.3 Data Collection

Previously trained researchers (3 Technicians of Psychiatric Rehabilitation and 2 Psychologists) have conducted the collection of data (by interviews and questionnaires). The staff has been contacted in departments over the course of the three shifts of work. People who decided to join signed an informed consent. The data collection took place in special rooms made available in each hospital ward or department by the medical head.

The administration of the tools has always been preceded by a short presentation of the research, its goals, and then proceed with a quick illustration of the brief structured interview and of the self-report questionnaires content; interviewers remained on site so as to provide answers to any questions or concerns, especially in order to avoid response bias in the fulfilling of self-administered questionnaires.

### 2.4 Research Instruments

1) The Maslach Burnout Inventory (MBI) is the most famous and international widely used instrument for the measurement of burnout and work-related psychopathology risk. The tool was developed by the group of Christina Maslach, Berkeley University; this study adopted the version for healthcare professionals [15], in the Italian version of Sirigatti and Stefanile [16]. The MBI consists of a questionnaire of 22 items, each with 6 points on a Likert scale from “never” to “every day.”

The MBI has been operationalized as composed of three components:

*Emotional/Psychophysical Exhaustion*. This dimension refers to the experience of feeling as emotionally drained and canceled by the work, a distressing condition (both physical and emotional), due to a perception of excessive job demands with respect to the personal resources, which is accompanied by emotional aridity in the relationship with others [17]

*Cynicism - Depersonalization*. This dimensions refers to an effective, although maladaptive, implementation of expulsion and rejection characterized by negative and rude behavioral responses to those (users in particular) that require or receive professional answers. It has been interpreted as a self-defense mechanism, aiming to minimize job involvement [18].

*Reduced personal accomplishment or efficacy* / *Lack of personal fulfillment*. This dimension refers to one's feelings of ineffectiveness, united to the collapse of self-esteem and the feeling of failure in the career and ideals of work [17].

2) Patient Health Questionnaire (PHQ-9). The PHQ-9 is a 9-item self-administered questionnaire aimed at assessing the presence of depressive symptoms during the previous two weeks (Spitzer et al 1999) [19]. As severity scores can range from 0 (absence of depressive symptoms) to 27 (severe depressive symptoms). Each of the 9 items specifically investigates each of the diagnostic criteria of DSM-IV [20], which for major depressive episode have remained the same in DSM-5 [21], and whose presence can be marked from 0 (not at all) to 3 (almost every day). Major depression is diagnosed if 5 or more of the nine criteria-symptoms were at least “more than half the days” (a score of 2) in the last two weeks, and one of the symptoms is depressed mood or anhedonia.

3) 12-Item Short Form Survey (SF-12). The quality of life (QoL) was measured with the SF-12 scale [22], which was already used in a large survey in the Italian version [23, 24]. The tool includes the following dimensions: physical health; limitations in activities due to the physical health; emotional state; physical pain; self-assessment of general health; vitality; social functioning and mental health. The observation period was the previous month. Higher scores correspond to a better Quality of Life. The instrument was designed to reduce burden for responders maintaining good standards of precision for purposes of large group comparisons.

4) Demographic and Work History Interview (SDL): a set of sixteen items provided a more accurate picture of the respondent's life. The following information were retrieved by directly asking about it: sex, age, educational level, marital status, the number of children, the type and the time of the labor contract system, the work department, the economic position according to the contract work, the years of total and those in the current administration work, previous work experience, the time taken to get to the workplace, the number of hours worked per day, and those extraordinary week on average. The socio-demographic part was the same used and validated for a broad epidemiological research in community [23].

### 2.5 Data Analysis

Data analysis aimed at identifying the frequency of psychopathological distress and / or impairment of quality of life in hospital workers and to identify the factors associated with distress and/or low quality of life. Specifically:

- a comparison between the average scores of the SF-12 (quality of Life) and PHQ-9 (screening for depression) questionnaires in our sample and in two samples coming from community surveys were investigated. The first sample involved over 2,000 representative interviews of the Italian national population. This study aimed to investigate the prevalence of mood disorders, the consumption of drugs and the quality of life. As for the assessment of positivity to screening for depression investigated with PHQ-9, the result reported in the sample of the present study was compared with a normative sample, from an already published survey, in which the PHQ-9 was administered to 1,500 subjects representing the Sardinian regional population [25].
  Univariate analysis was carried out on the average SF-12 and PHQ-9 score as the dependent variable. It was conducted by comparing the unweigh averages of the sample of health workers against each of the other samples as well as after indirect standardization of the two control samples, taking into consideration the distribution by sex and age in the worker sample. It has been considered in this regard four cells obtained after subdivision by age and gender (the subdivision by age was given by having 40 years or less or more than 40 years).
- a multivariate logistic regression analysis to evaluate the effect of different independent variables on the probability of being positive, respectively, at least two sub-scales of the Maslach Burnout Inventory (cut-off: EE> = 24, DP> = 9 and / or RP = <29), to obtain an SF-12 lower than the average national score - a standard error (> 36), as well as to be detected positive at the screening PHQ-9 for depressive episodes (> 8). The independent variables considered in the analysis were: sex (male), age (<= 40 years), education, marital status, and work in a specific hospital department (*e.g.* cardiology or urgency-emergency wards).

### 2.6 Ethical Standards

The ethics committee of the Azienda Ospedaliero Universitaria di Cagliari approved the study project (protocol n° NP/2015/2824, May 26^th^, 2015). The study protocol complies with the Declaration of Helsinki and its revisions [26].

Research had been agreed with the General Directorate of the agency. The researchers have declared they were willing to provide and discuss the research results with management and with the heads of work medicine of the agency. A similar commitment was also included in the request to the Ethics Committee of the Agency. Informed consent was obtained from the people who have agreed to take part in the project.

## 3 RESULTS

Table **1** shows the characteristics of the interviewed sample. Overall, 522 workers accepted to take part in the study, representing a 78% response rate (669 individuals).

There were 147 subjects (women: n = 87; 59%) who refused to participate or were not tracked because on vacation or have leaved work for long sickness. The distribution by age and sex of those who were not interviewed did not differ from the one of those who agreed to participate.

Women were 66.3% of the interviewed samples. Most were 40 years old or older (64.8%). Graduates represent around a half of the sample as did married / living with a partner and those who didn't have children.

The table also shows the time spent by subjects to complete the questionnaires and to answer to the interview: the 48.9% of the sample took less than 15 minutes, with a mean in the sample of 14.7 ± 8.9 minutes.

Table **1** illustrates the working employment contract of the sample. A significant portion of the sample (24.9%) work under a fixed-term contract, the vast majority is employed full-time (95.8%) and only a small portion was administrative staff. Only 36.3% earn 2,500 euros or more, and more than 40% worked from more than 20 years.

Table **2** compares the demographic variables of the interviewed sample with those of the two samples of the general population that serve as “normative” standard. The sample of this research is not representative of the adult population, and it is in fact a sample of older on average workers, almost 65% has more than 40 years and 33% have more than 50 (Table **2**).

In addition, women were over-represented compared to the community samples.

A first confrontation without balancing highlights a frightening state of malaise in the study sample (Table **2**).

The frequency of depressive symptoms (positive at PHQ-9) is indeed the 33.2% in the examined health workers against the 13.1% of the community sample (OR = 4.60; 95% CI 3.57-5.92; P <0.0001), while the frequency of individuals who accuse a low level of quality of life (below the national average minus one standard error) was 42.7% against 23.6% of the general population (OR = 2.40; Cl95% 1.96-2.94; P <0.0001).

The comparison made after indirect standardization by age and sex of the two normative samples confirms the state of malaise in the sample of workers with risk of low quality of life (47% in the study sample versus 25.9% in the “normative” sample, OR = 2.18; Cl95% 1.78-2.67) and positivity screened for depression (33.2% against 14.1% in the community sample, OR = 2.89; Cl95%).

Table **3** examines the factors associated with having a depressive episode (*i.e.*, to be positive on the PHQ-9). To be male, and in youthful age were protective factors.

Those who were older and working in the University Hospitals had a risk that was not the simple sum of the two risk factors but they were amplified each other.

As for the risk related to the work in specific departments the “surgery” department only was associated with a higher risk, while working in laboratories was a protective factor.

Table **4** leads to the same type of analysis by professions: all workers of the university hospitals of all professions show a risk of developing a depressive episode higher than the regional normative sample except for technical and administrative staff.

Table **5** analyzes the quality of life in our sample that is combined for analysis at the national normative sample. The analysis was conducted using which cut-off the average of the national normative sample less a standard error calculated for a sample of the size of the one examined.

Males and less of 40 years old workers of both sexes showed a better quality of life. Also, examining work at the company is not a factor with respect to the risk of interaction by gender and age. Being a woman, more than 40 years old and working at the health care agency are risk factors that add without interaction (not amplify each other).

As shown in Table **6**, is associated with a low quality of working life in the surgical wards; in Cardiology, Ophthalmology, Oncology (P <0.001) and Dermatology (P <0.05).

Table **7** shows the results concerning the risk of burn-out within the sample of this survey. The analysis has been conducted by logistic regression multivariate analysis with, as dependent variable, being positive to at least two of the three factors of the MBI questionnaire and as independent variables the demographic conditions (age and sex), the work history and work in specific departments. The work in the surgical wards is the only factor found to expose to the risk.

Table **8** analyzes the antecedents of burnout (positivity to at least two scales of MBI), subtracting in the multivariate analysis the kind of department / ward as an independent variable and introducing the type of work task and positivity at PHQ-9 and SF-12 (with the same cut-off already utilized). The Health Assistants have found to be protected from this risk. Also, the results highlights that score at SF-12 was negatively related to the risk of burnout (the highest score on this scale indicates a good level of quality of life) while in contrast the score of 8 or less at PHQ-9 is directly proportional. *i.e.*, as better the quality of life, as lower the risk of burnout and, conversely, as higher the levels of depression, as higher the risk of burnout.

## 4 DISCUSSION

The present study found that in the overall health care workers sample, the frequency of positivity at the screener for Major Depressive Disorder is more than double of levels in the standardized community sample (33.3% vs 14.1%, p<0.0001). All the professionals, except the administrative staff and technicians (*i.e.* those who do not have contact with patients), showed a statistically higher frequency of individuals screened positive for depressive episode compared to the representative sample of the population of the region of Sardinia. Furthermore, being older than 40 years is not a risk factor for the normative regional control sample, but an interaction was found between being older than 40 years and working in the studied agency, with an amplification of the effect risk between the two conditions (age and working in the university hospitals). Results are in line with previous studies [25], which highlighted the protective factors of being a male and in youthful age. Furthermore, considering the specific work environment, results showed that working in surgery department is associated with a higher risk, while working in laboratories is a protective factor. These results are in line with previous researches that showed how distress, including anxiety, depression, alcoholism, substance abuse, are more prevalent among surgeons [27].

Past studies showed that being positive to the PHQ-9 is associated to a depression diagnosis. It is estimated that between 30% and 50% of positives to the PHQ-9 are clinically depressed [28, 29]. Considering our results, we found that positivity to the screening is very high among public health workers population (rate of 33%) while among the general population it is 13%. Taking into account that positivity to PHQ-9 corresponds to a clinical diagnosis of depression in about one third/fourth of cases, prevalence rates of clinical depression among public health workers and general population in these samples can be estimated respectively 8% and 4%.

Almost all of the professionals who carry out their work at the two university hospitals of a public Italian university care agency showed a risk of developing a depressive episode higher than the regional normative, and reported low level of perceived quality of life. These frequencies are higher, with statistically significant differences, with respect to a national representative sample of the population even after standardization by age and sex. The only exceptions are represented by the administrative staff and by technicians. Depression is a common mental disorder, with a prevalence of 14.6% among adults in high-income countries and 11.1% in developing countries [30]. Our results are in line with many studies that showed that the prevalence of depressive symptoms among health-care workers ranged from 18% to 41%.

Finally, considering the quality of working life, results are in line with the research literature. Health care workers are constantly exposed to the risk of low quality of working life. We did no find any difference when we considered profession and working unit. The quality of working life is considered as a factor beyond job satisfaction and is related to personnel’s well-being [14, 31]. In this sense, international studies showed how health-care workers (mainly nurses and physicians) were low in their quality of working life. Researchers mentioned that the health-care working context is inherently stressful [32], and many of the health-care workers are exhausted [33, 34].

When we considered job burnout levels, we found high burnout levels in the surgery unit. Results confirmed that incidence of burnout among health care workers affects more than the 30% of workers globally [35], reaching rates between 25% and 75% in some clinical specialties [36]. Specifically, among surgeons it has been reported reaching rates of 28% to 42% [27, 37, 38]. Burnout is very common among physicians if compared with depression and substance abuse. Considered as a clinical syndrome, burnout is characterized by emotional exhaustion, depersonalization, and a decreased sense of personal accomplishment. Surgeons’ burnout is linked to patient’s safety and quality of patient’s care, and contributes to medical errors [8, 9].

The highest frequency of positive screening for depression and lower quality of working life registered in the surgeon unit is of particular concern. Surgical practice is characterized by hard work, working long hours, dealing regularly with life-and-death environment with patients, and sometimes the sacrifice of personal life to the practice in the field [26]. According to the research literature, to reduce surgeon’s distress and then the risk of burnout, intervention programs should be aimed at reducing worker’s experience of stressors such as reducing workers’ workload, increasing their sense of control and promoting organizational health.

In sum, this study aimed to make a contribution towards public sector management, mostly considering the central role played by workplace characteristics that can improve employee well-being in public hospitals.

The present study has some limitations. First, a convenience sample has been used. This can limit the generalizability of the results, reducing external validity of the study. Another limitation is represented by the use of a self-reported questionnaire, which may yield a bias related to social desirability and common method bias. Finally, this study includes a cross-sectional design type and we are unable to examine the causal effect of the relationship between variables. This effect would be better analyzed through longitudinal studies, which would add something more about the development of mental health in working context.

## Figures and Tables

**Table 1:** Demographic characteristics of the interviewed sample and risk factors.

**Age**	** N**	** %**
≤ 30	86	16.86
31-40	97	18.82
41-50	157	30.78
> 50	171	33.53
**Gender**		
Female	337	66.08
Male	173	33.92
**Education**		
Primary School	31	5.9
Secondary School	128	24.5
University Degree	73	14.0
At least Fire Years of University Courses	289	55.4
**Marital Status**		
Single never married	205	40.20
Separated / Divorced	49	9.61
Married / Living together	250	49.02
Widow	6	1.18
**Number of sons**		
0	252	49.41
1	92	18.4
≥2	166	32.55
**Time for interview and questionnaires (minutes)**		
<15’	256	48.9
16-30	205	51.1
**Kind of Contract**		
Open-ended contract	380	74.71
Fixed-term contract	130	25.49
**Working Time**		
Full-time	489	95.88
Part-time	21	4.12
**Professional Sector**		
Management / Administrative	11	2.11
Health without constant direct contact with Patients	313	60.19
Health with constant direct contact with Patients	196	37.69
**Professional Role**		
Medical Doctor	192	36.78
Nurse	207	39.81
Healthcare Assistant	62	11.92
Nurse coordinator	14	2.69
Technician	33	6.35
Social Coordinator or Management/Administration Staff	14	2.69
**Net Salary (Euro)**		
Less than 1,500	74	14.51
1,500-2,500	247	48.43
>2,500	189	37.06
**Total Years at work**		
≤5	125	24.51
6-10	65	12.75
11-20	113	22.16
21-30	141	27.65
>30	66	12.94
**Time spent to joint work from house**		
≤15’	248	48.63
16’-30’	204	40.00
>30’	58	11.37
**Paid overtime in a week**		
None	260	49.7
1-5h	176	33.7
>5h	87	16.6

**Table 2:** Comparison by univariate analysis between the sample study and two community normative samples.

	Research Sample	Normative Sample SF-12*	Comparison	Normative Sample PHQ-9**	Comparison
**Age** 18-30	85 (16.3%)	540 (23.1%)	Χ2=11.62 P<0.001	302 (20.15)	Χ2= 3.59 P=0.049
31-40	98 (18.85)	386 (16.6%)	Χ2=1.07 P=0.30	255 (17.0%)	Χ2=0.59 P=0.440
41-50	161 (31.0%)	416 (18.0%)	Χ2=45.02 P<0.0001	272 (18.1%)	Χ2=37.91 P<0.0001
> 50	176 (33.8%)	985 (42.2%)	Χ2=73.81 P<0.0001	673 (44.8%)	Χ2=14.75 P<0.0001
**Gender** Female	346 (66.3%)	1320 (56.9%)	Χ2=375.3 P<0.0001	774 (51.5%)	Χ2=228.7 P<0.0001
Score SF-12 ≤<36	222 (42.7%)	551 (23.6%)	X2=78.2,P<0.0001 OR=2.40; Cl95% 1.96-2.94	//	//
Score SF-12 < 36 (after indirect standardization)	222 (42.7%)	606 (25.9%)	X2=67.76, P<0.0001 OR=2.18; Cl95% 1.78-2.67	//	//
Score PHQ-9 >8	173 (33.2%)	//	//	197 (13.1%)	X2=164,8 P<0.0001 OR=4.60; Cl95% 3.57-5.92
Score PHQ-9 >8 (after indirect standardization)	173 (33.3%)	//	//	221 (14.1%)	X2=85,07 P<0.0001 OR=2.89; Cl95% 2.89-3.66

*Carta *et al.* 2012 ** Moro *et al.* 2015

**Table 3:** Risk for Depressive Episodes in the study sample (positives at PHQ-9). Independent determinants general demographic variables and work in specific departments.

**Dependent Variable: positivity at PHQ-9 (>8)**			**Coef.**		**Robust standard error**	**Marginal** **Effects**		**Robust standard error**
Gender (male)			-0,892	***	0,165	-0,115	***	0,020
Age (18-30)			-0,431	*	0,254	-0,052	*	0,028
(41-50)			-0,404		0,262	-0,049	*	0,029
(50-70)			-0,205		0,207	-0,027		0,027
Wards of General Medicine Hosp A			0,630		0,411	0,101		0,078
Wards of General Medicine Hosp B			-0,143		0,439	-0,018		0,053
Wards of Surgery			0,880	**	0,389	0,151	*	0,082
Endoscopy and - Radiology			0,368		0,474	0,055		0,079
Laboratory			-1,809	**	0,788	-0,131	***	0,025
Intensive Care Unit			0,225		0,412	0,032		0,063
Dermatology			-0,167		0,632	-0,021		0,075
Cardiology			0,701		0,434	0,115		0,085
Ophthalmology			0,676		0,459	0,111		0,089
Oncology			0,624		0,489	0,101		0,093
Neurology			0,422		0,676	0,064		0,116
Maternity Hospital			0,259		0,532	0,037		0,083
Neonatology			0,378		0,411	0,057		0,069
Emergency			0,108		0,514	0,015		0,073
***Interactions***								
Gender (male) x working in the health agency			0,463	*	0,277	0,070		0,046
Age (18-30) x working in the health agency			0,119		0,445	0,016		0,064
Age (41-50) x working in the health agency			1,138	***	0,392	0,202		0,086
Age (50-70) x working in the health agency			0,760	**	0,363	0,123		0,070
Log-Likelihood function	-867,5787							
Pseudo-R-squared	0,0894							
Valid Observations	2012							

*<p<0.10, P<0.05**, P<0.01***

**Table 4:** Risk for Depressive Episodes in the study sample (positives at PHQ-9). Independent determinants general demographic variables and kind of work in specific departments.

**Dependent Variable: positivity at PHQ-9 (>8)**		**Coef.**		**Robust standard error**
Executive Health Care Role		0,647	***	0,194
Nurse		1,479	***	0,166
Healthcare Assistant		1,228	***	0,284
Nurses Coordinator		1,091	**	0,552
Technician		0,645		0,433
Administrative		1,710		1,292
Log-Likelihood function	-878,846			
Pseudo-R-squared	0,078			
Number of Effective Observations	2012			
*<p<0.10, P<0.05**, P<0.01***				

**Table 5:** Quality of life in the study sample and in the national normative sample (Cut-off = mean – 1 standard error in the normative sampe = 36). Independent determinants: general demographic variables and work in specific departments.

**Dependent Variable: mean – 1 standard error in the normative sampe score = 36**		**Robust standard error**	**Coef.**		**Marginal** **Effects**	
Gender (male)		0,617	***	0,107	0,111	***
Age (18-30)		0,251		0,196	0,045	
(41-50)		-0,360	*	0,187	-0,070	*
(50-70)		-1,040	***	0,159	-0,201	***
Wards of General Medicine Hosp A		-0,851	**	0,412	-0,186	*
Wards of General Medicine Hosp B		-0,627		0,422	-0,133	
Wards of Surgery		-1,435	***	0,382	-0,330	***
Endoscopy and - Radiology		-0,776	*	0,446	-0,168	
Laboratory		0,612		0,531	0,096	
Intensive Care Unit		-0,384		0,407	-0,078	
Dermatology		-1,210	**	0,532	-0,276	**
Cardiology		-1,196	***	0,421	-0,272	***
Ophthalmology		-1,633	***	0,454	-0,379	***
Oncology		-1,361	***	0,475	-0,313	***
Neurology		-0,587		0,560	-0,124	
Maternity Hospital		-0,307		0,585	-0,061	
Neonatology		-0,745	*	0,389	-0,160	*
Emergency		-0,465		0,504	-0,096	
***Interactions***						
Gender (male) x working in the health agency		0,186		0,248	0,033	
Age (18-30) x working in the health agency		0,047		0,406	0,009	
Age (41-5) x working in the health agency		-0,633	*	0,349	-0,133	
Age (>50) x working in the health agency		0,055		0,342	0,010	
Log-Likelihood function	-1504,084					
Pseudo-R-squared	0,088					
Osservazioni	2834					
*<p<0.10, P<0.05**, P<0.01***						

**Table 6:** Risk of low level of Quality of Life in the study sample (Cut-off = mean – 1 standard error in the normative sample = 36). Independent determinants professional role.

**Dependent Variable: mean – 1 standard error in the normative sampe score = 36**		Coef.		Robust standard error
Executive Health Care Role		-0,607	***	0,176
Nurse		-1,290	***	0,148
Healthcare Assistant		-0,671	**	0,277
Nurses Coordinator		-1,397	**	0,554
Technician		-0,272		0,384
Administrative		-1,085		1,086
Log-Likelihood function	-1515,915			
Pseudo-R-squared	0,082			
Osservazioni	2836			
Nota: *,**, *** alpha threshold at, respectively, p = 0.10, 0.05, and 0.01				

**Table 7:** Determinants of positivity to at least two subscales of the Maslach Burnout Inventory.

**Dependent Variable: positivity to at least two subscales of the Maslach Burnout Inventory**	**Coef.**		**Robust standard error**	**Effetti marginali**			**Robust standard error**
Gender (male)	-0,270		0,297	-0,028			0,029
Age (<40 years)	-0,428		0,496	-0,044			0,049
Education (max 8 years)	0,889	*	0,512	0,127			0,092
Education (max 13 years)	0,245		0,333	0,028			0,039
Married / Living Together	-1,061	*	0,603	-0,083	**		0,033
Separated / Divorced	-0,403		0,302	-0,043			0,033
Widow	0,847		0,832	0,122			0,151
1/0 sons	-0,289		0,400	-0,029			0,037
Total years at work (>14)	-0,310		0,485	-0,034			0,054
Wards of General Medicine Hosp A	2,051	*	1,074	0,367			0,247
Wards of General Medicine Hosp B	0,388		1,195	0,047			0,160
Wards of Surgery	2,491	**	1,065	0,468	**		0,240
Endoscopy and - Radiology	1,090		1,153	0,162			0,220
Laboratory	0,468		1,294	0,058			0,185
Intensive Care Unit	0,883		1,123	0,122			0,191
Dermatology	1,000		1,245	0,148			0,236
Cardiology	0,540		1,205	0,069			0,178
Ophthalmology	1,902	*	1,113	0,344			0,263
Oncology	0,996		1,191	0,146			0,222
Neurology	1,103		1,248	0,170			0,250
Maternity Hospital	1,662		1,078	0,277			0,236
Neonatology	1,591		1,134	0,272			0,257
Emergency							
Log-Likelihood function	194,291						
Pseudo-R-squared	0,095						
Number of Effective Observations	510						
*<p<0.10, P<0.05**, P<0.01***							

**Table 8:** Risk od burnout in the study sample (**positivity to at least two subscales of the Maslach Burnout Inventory)**. Independent determinants: low qaulaity of life; depressive episode and professional role.

**Dependent Variable: positivity to at least two dimensions of the Maslach Burnout Inventory.**	**Coef.**	**Robust standard error**	
SF-12 (>36)	-1,795	***	0,427
PHQ-9 (>8)	1,577	***	0,359
Executive Health Care Role	-1,420		0,932
Nurse	-1,182		0,890
Healthcare Assistant	-1,867	**	0,940
Nurses Coordinator	-1,365		1,163
Technician and administrative	-0,979		1,036
Log-Likelihood function	-158,072		
Pseudo-R-squared	0,257		
N of observations	508		
*<p<0.10, P<0.05**, P<0.01***			
